# Two major metabolic factors for an efficient NADP-malic enzyme type C_4_ photosynthesis

**DOI:** 10.1093/plphys/kiac051

**Published:** 2022-02-15

**Authors:** Honglong Zhao, Yu Wang, Ming-Ju Amy Lyu, Xin-Guang Zhu

**Affiliations:** Center of Excellence for Molecular Plant Sciences, Chinese Academy of Sciences, Shanghai 200032, China; The Carl R. Woese Institute for Genomic Biology, University of Illinois Urbana-Champaign, Champaign, Illinois 61801, USA; Center of Excellence for Molecular Plant Sciences, Chinese Academy of Sciences, Shanghai 200032, China; Center of Excellence for Molecular Plant Sciences, Chinese Academy of Sciences, Shanghai 200032, China

## Abstract

Compared to the large number of studies focused on the factors controlling C_3_ photosynthesis efficiency, there are relatively fewer studies of the factors controlling photosynthetic efficiency in C_4_ leaves. Here, we used a dynamic systems model of C_4_ photosynthesis based on maize (*Zea mays*) to identify features associated with high photosynthetic efficiency in NADP-malic enzyme (NADP-ME) type C_4_ photosynthesis. We found that two additional factors related to coordination between C_4_ shuttle metabolism and C_3_ metabolism are required for efficient C_4_ photosynthesis: (1) accumulating a high concentration of phospho*enol*pyruvate through maintaining a large PGA concentration in the mesophyll cell chloroplast and (2) maintaining a suitable oxidized status in bundle sheath cell chloroplasts. These identified mechanisms are in line with the current cellular location of enzymes/proteins involved in the starch synthesis, the Calvin–Benson cycle and photosystem II of NADP-ME type C_4_ photosynthesis. These findings suggested potential strategies for improving C_4_ photosynthesis and engineering C_4_ rice.

## Teaser

High levels of PGA and PEP in mesophyll cell chloroplasts and a suitable oxidation state in bundle sheath cell chloroplasts are the requirements for efficient C_4_ photosynthesis.

## Introduction

With the global population growth, improved economic status, and changing climate, securing food supply is still a major issue facing our society. Increasing productivity of both C_3_ and C_4_ crops is needed to realize the desired food security (Ray et al., 2013; Hunter et al., 2017). Much effort has been made to improve photosynthetic efficiency of C_3_ plants (Long et al., 2015; Shen et al., 2019; South et al., 2019; López-Calcagno et al., 2020). Comparatively, the efforts to improve photosynthetic efficiency in C_4_ plants are lacking.

To increase photosynthesis in C_4_ plants, some individual enzymes limiting C_4_ photosynthesis have been identified by calculating the control coefficient of each enzymatic step. For example, carbonic anhydrase (CA; von Caemmerer et al., 2004; Studer et al., 2014), phosphoenolpyruvate carboxylase (PEPC; Boyd et al., 2015), and pyruvate orthophosphate dikinase (PPDK; Wang et al., 2008) may have relatively large control coefficients on the C_4_ photosynthetic CO_2_ fixation rate (*A*; von Caemmerer and Furbank, 2016). Additionally, Rubisco is identified as a key limiting factor for C_4_ photosynthesis (von Caemmerer et al., 1997; Wang et al., 2014a, 2014b). Salesse-Smith and collaborators increased Rubisco activity in maize (*Zea mays*) by the overexpression of Rubisco subunits together with the assembling chaperone RUBISCO ASSEMBLY FACTOR 1, which results in increased photosynthesis and biomass in transgenic maize compared with those in the wild-type (WT) (Salesse-Smith et al., 2018). All these studies show that there is room to further enhance the photosynthetic CO_2_ uptake rate in C_4_ plants (Matsuoka et al., 2001; von Caemmerer and Furbank, 2016).

Besides improving the photosynthetic efficiency of C_4_ plants, great efforts have been invested into engineering C_3_ plants to perform C_4_ photosynthesis. The C_4_ plants usually have higher photosynthetic energy conversion efficiency compared to C_3_ plants, as a result of having a CO_2_ concentration mechanism (CCM; Zhu et al., 2008), which functions as a CO_2_ “pump,” that is, concentrating CO_2_ around Rubisco in the bundle sheath cells (BSCs) and inhibiting photorespiration (Sage, 2004). Introducing the CCM of C_4_ photosynthesis into C_3_ crops through genetic engineering can potentially enhance yield of C_3_ crops by 50% (Matsuoka et al., 2001; Hibberd et al., 2008; Covshoff and Hibberd, 2012; Furbank et al., 2015). Historically, hybridization between C_3_ and C_4_ plants has been used as a strategy to realize this goal, as summarized by Brown and Bouton (1993). Unfortunately, this effort did not succeed largely due to the difficulty of obtaining stable genetically inheritable germplasm after hybridization. Later, single enzymes of C_4_ photosynthesis and multigene combinations for the NADP-malic enzyme (NADP-ME) type C_4_ CCM were introduced into rice (*Oryza sativa* ; Fukayama et al., 2001, 2003, 2006; Tsuchida et al., 2001; Taniguchi et al., 2008), which culminated in the incorporation of a single-cell C_4_ photosynthetic pathway (Miyao et al., 2011) or the two-cell NADP-ME type C_4_ photosynthetic pathway into rice (Ermakova et al., 2020). Unfortunately, so far, in transgenic plants with enhanced expression of CCM-related enzymes, photosynthesis did not increase (Ku et al., 2001; Miyao et al., 2011; Ermakova et al., 2020). Two recent reports showed that a partial NADP-ME type C_4_ photosynthetic biochemical pathway has been successfully constructed in rice (Lin et al., 2020; Ermakova et al., 2021). The enzymes simultaneously overexpressed in the rice plants include PEPC, PPDK, NADP-ME, and malate dehydrogenase (Lin et al., 2020). Additionally, a two-cell prototype by overexpressing the four C_4_ cycle enzymes above and CA in rice with the specific cellular compartmentations has also been developed (Ermakova et al., 2021). In both studies, a partial C_4_ biochemical pathway has been successfully installed (Lin et al., 2020; Ermakova et al., 2021). Since there are no remarkable malate decarboxylation fluxes detected in their work, the whole cycle of C_4_ CCM in transgenic rice seems unfunctional.

The lack of detectable C_4_ fluxes implies that there might be other major features that are missed in the current C_4_ engineering effort. The lack of a complete C_4_ cycle in the engineered plants might be due to a lack of the sufficient metabolite transporter activities, existence of barriers preventing CO_2_ leakage from BSCs to mesophyll cells (MCs), and a lack of a sufficient number of chloroplasts in BSC (Bchl; Lin et al., 2020). A number of recent studies suggest some metabolic features which may be equally important for the operation of a complex metabolic pathway. For example, maintaining sufficiently high concentrations of the intermediates is a major factor to ensure a sufficiently high metabolite flux through a system (Antonovsky et al., 2016; Barenholz et al., 2017). Furthermore, to ensure a highly efficient system, simple over-expression of enzymes in the metabolic pathway is not sufficient. For example, overexpressing transketolase in the Calvin–Benson cycle (CBC) leads to the disruption of the metabolic coordination for triose phosphate (T3P) available for different metabolism pathways, ultimately inhibiting plant growth (Khozaei et al., 2015). Our recent theoretical analysis shows that the excess enzymatic capacity of some steps in the CBC can reduce metabolic network efficiency as a result of an imbalanced distribution of shared metabolites among different sub-cycles (Zhao et al., 2020). Considering that C_4_ photosynthesis requires highly efficient coordination between BSC and MC (Stitt and Zhu, 2014), the question arising here is: are there metabolic features that are required for C_4_ photosynthesis that have been missed in all current C_4_ engineering efforts?

This study aims to identify the metabolic features required to gain an efficient C_4_ photosynthesis, which will not only be crucial for the current efforts of improving photosynthetic efficiency of contemporary C_4_ plants, but also provide important targets for consideration in the current efforts of engineering C_3_ plants to perform C_4_ photosynthesis.

## Results

### Model analysis

Although CO_2_ is initially fixed by PEPC in C_4_ photosynthesis, it is ultimately fixed by Rubisco. In other words, the C_4_ photosynthetic rate can be calculated based on the reaction catalyzed by Rubisco. Net photosynthetic CO_2_ fixation rate (*A*) is calculated by the following formula (Farquhar et al., 1980):

(1) $$A=v_{c}\;-\;0.5\;*\;v{}_{o}-Rd\ldots$$

where *v*_c_ and *v_o_* represent the rates of RuBP (ribulose -1,5- bisphosphate) carboxylation and oxygenation catalyzed by Rubisco, respectively. *Rd* represents the CO_2_ release rate from day respiration. Its value is assumed to be 1 *μ*mol m^−2^ s^−1^ as in the previous model (Wang et al., 2014a, 2014b). According to the Michaelis–Menten kinetics, the *v*_c_ and *v_o_* are described as (Wang et al., 2014a, 2014b):

... (2) $$v_{c}=V_{\mathrm{cmax}}\;*\frac{{\left[\mathrm{RuBP}\right]}_{\mathrm{Bchl}}}{{\left[\mathrm{RuBP}\right]}_{\mathrm{Bchl}}+KM_{\mathrm{RuBP}}}*\frac{{\left[CO_{2}\right]}_{\mathrm{Bchl}}}{{\left[CO_{2}\right]}_{\mathrm{Bchl}}+KM_{CO_{2}}*(1+\frac{{\left[O_{2}\right]}_{\mathrm{Bchl}}}{Ki_{O_{2}}})}$$

 

... (3) $$v_{o}=V_{\mathrm{omax}}\;*\frac{{\left[\mathrm{RuBP}\right]}_{\mathrm{Bchl}}}{{\left[\mathrm{RuBP}\right]}_{\mathrm{Bchl}}+KM_{\mathrm{RuBP}}}*\frac{{\left[O_{2}\right]}_{\mathrm{Bchl}}}{{\left[O_{2}\right]}_{\mathrm{Bchl}}+KM_{O_{2}}*\left(1+\frac{{\left[{\mathrm{CO}}_{2}\right]}_{\mathrm{Bchl}}}{Ki_{{\mathrm{CO}}_{2}}}\right)}$$

 

(4) $$V_{\mathrm{omax}}=0.11\;*\;V_{\mathrm{cmax}}\ldots$$

where *V*_max_ is the maximal velocity of Rubisco, KM_RuBP_ is the apparent Michaelis–Menten constant of Rubisco for RuBP; KM_CO2_ and KM_O2_ are the Michaelis–Menten constants of Rubisco for CO_2_ and O_2_, respectively; Ki_CO2_ and Ki_O2_ are the inhibition constants of CO_2_ and O_2_, respectively; the subscript “Bchl” means chloroplast in the BSC.

According to Equations (1–4),

(5) $$A=V_{\mathrm{cmax}}\;*\frac{{\left[\mathrm{RuBP}\right]}_{\mathrm{Bchl}}}{{\left[\mathrm{RuBP}\right]}_{\mathrm{Bchl}}+KM_{\mathrm{RuBP}}}*\left(\frac{{\left[CO_{2}\right]}_{\mathrm{Bchl}}}{{\left[CO_{2}\right]}_{\mathrm{Bchl}}+KM_{CO_{2}}*\left(1+\frac{{\left[O_{2}\right]}_{\mathrm{Bchl}}}{Ki_{O_{2}}}\right)}\;-\frac{0.11*{\left[O_{2}\right]}_{\mathrm{Bchl}}}{{\left[O_{2}\right]}_{\mathrm{Bchl}}+KM_{O_{2}}*\left(1+\frac{{\left[{\mathrm{CO}}_{2}\right]}_{\mathrm{Bchl}}}{Ki_{{\mathrm{CO}}_{2}}}\right)}\right)-\mathrm{Rd}\ldots$$

We define that:

(6) $$f\left(\left[\mathrm{RuBP}\right]\right)=\;\frac{{\left[\mathrm{RuBP}\right]}_{\mathrm{Bchl}}}{{\left[\mathrm{RuBP}\right]}_{\mathrm{Bchl}}+KM_{\mathrm{RuBP}}}\ldots$$

 

(7) $$f\left(\left[CO_{2}\right]\right)=\frac{{\left[CO_{2}\right]}_{\mathrm{Bchl}}}{{\left[CO_{2}\right]}_{\mathrm{Bchl}}+KM_{CO_{2}}*\left(1+\frac{{\left[O_{2}\right]}_{\mathrm{Bchl}}}{Ki_{O_{2}}}\right)}\;-\frac{0.11*{\left[O_{2}\right]}_{\mathrm{Bchl}}}{{\left[O_{2}\right]}_{\mathrm{Bchl}}+KM_{O_{2}}*\left(1+\frac{{\left[{\mathrm{CO}}_{2}\right]}_{\mathrm{Bchl}}}{Ki_{{\mathrm{CO}}_{2}}}\right)}\ldots$$

**Figure 1:**
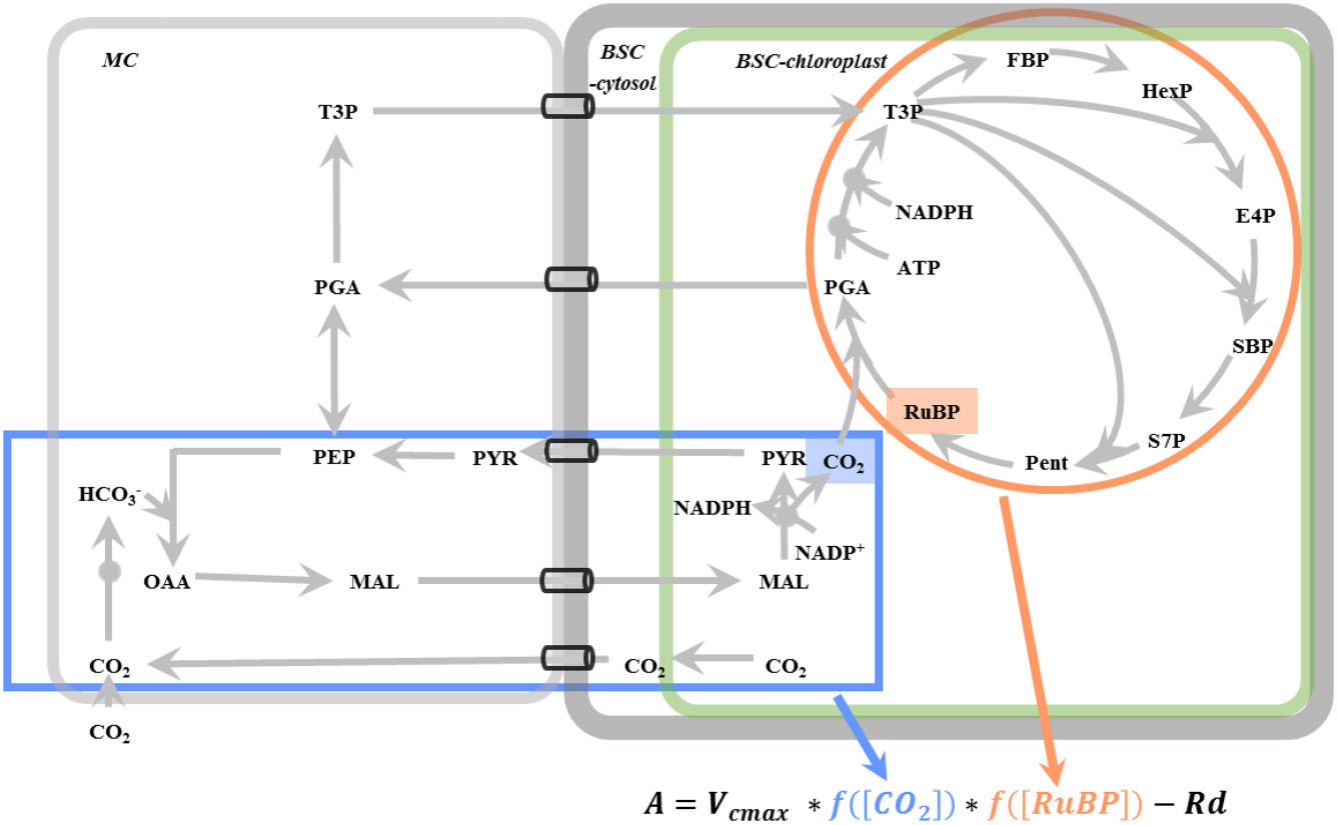
A simplified metabolic diagram of NADP-ME type C_4_ photosynthesis. CO_2_ is ultimately fixed by Rubisco carboxylation. Under a photosynthetic photon flux density of 1,500 μmol m^−2^ s^−1^, a CO_2_ concentration of 800 ppm and an O_2_ level of 21%, the net photosynthetic CO_2_ fixation rate (*A*) is determined by the concentrations of CO_2_ (blue square) and RuBP (orange square) around Rubisco in the Bchl. The operation efficiencies of C_4_ cycle (metabolic module in the blue box) and CBC (metabolic module in the orange circle) determine the efficiencies of CO_2_ concentrating and RuBP generation, respectively. *V*_max_ is the maximal velocity of Rubisco. *f([RuBP])* and *f([CO_2_])* are the functions shown in Equations (6) and (7), respectively. *Rd* means the dark respiration rate.

Such that *A* is calculated as (Figure 1):

(8) $$A=V_{\mathrm{cmax}}\;*f\left(\left[\mathrm{RuBP}\right]\right)*f\left(\left[CO_{2}\right]\right)-\mathrm{Rd}\ldots$$

In this study, we aimed to study the inherent biochemical requirements to gain an efficient NADP-ME type C_4_ photosynthesis. So we conducted the simulations under both high-light and high-CO_2_ level where there is no limitation of either light or CO_2_. Specifically, we assume an irradiance of 1500μmol m^−2^ s^−1^, an O_2_ levels of 21% and a [CO_2_] of 800 ppm. The rate of the Rubisco catalyzed reaction is determined by maximal Rubisco carboxylation capacity, the concentrations of RuBP, CO_2_, and O_2_ concentrations around Rubisco (Equation (8)). Based on the transformed Michaelis–Menten equation ($\frac{v}{V\max}=\frac{\frac{\left[S\right]}{Km}}{\frac{\left[S\right]}{Km}+1}$), the relationship between enzyme and its substrates ($\frac{v}{V\max}$ versus $\frac{\left[S\right]}{Km}$) is proposed as a measure of the limiting status for an enzymatic reaction (Fendt et al., 2010; Zhao et al., 2020). We used the same method as in Zhao et al. (2020) to identify enzymes which show biphasic responses to changing enzyme activities and study the underlying metabolic basis of the biphasic responses.

### Increasing the enzymatic capacity related to C_3_ metabolism may inhibit NADP-ME type C_4_ photosynthesis

**Figure 2:**
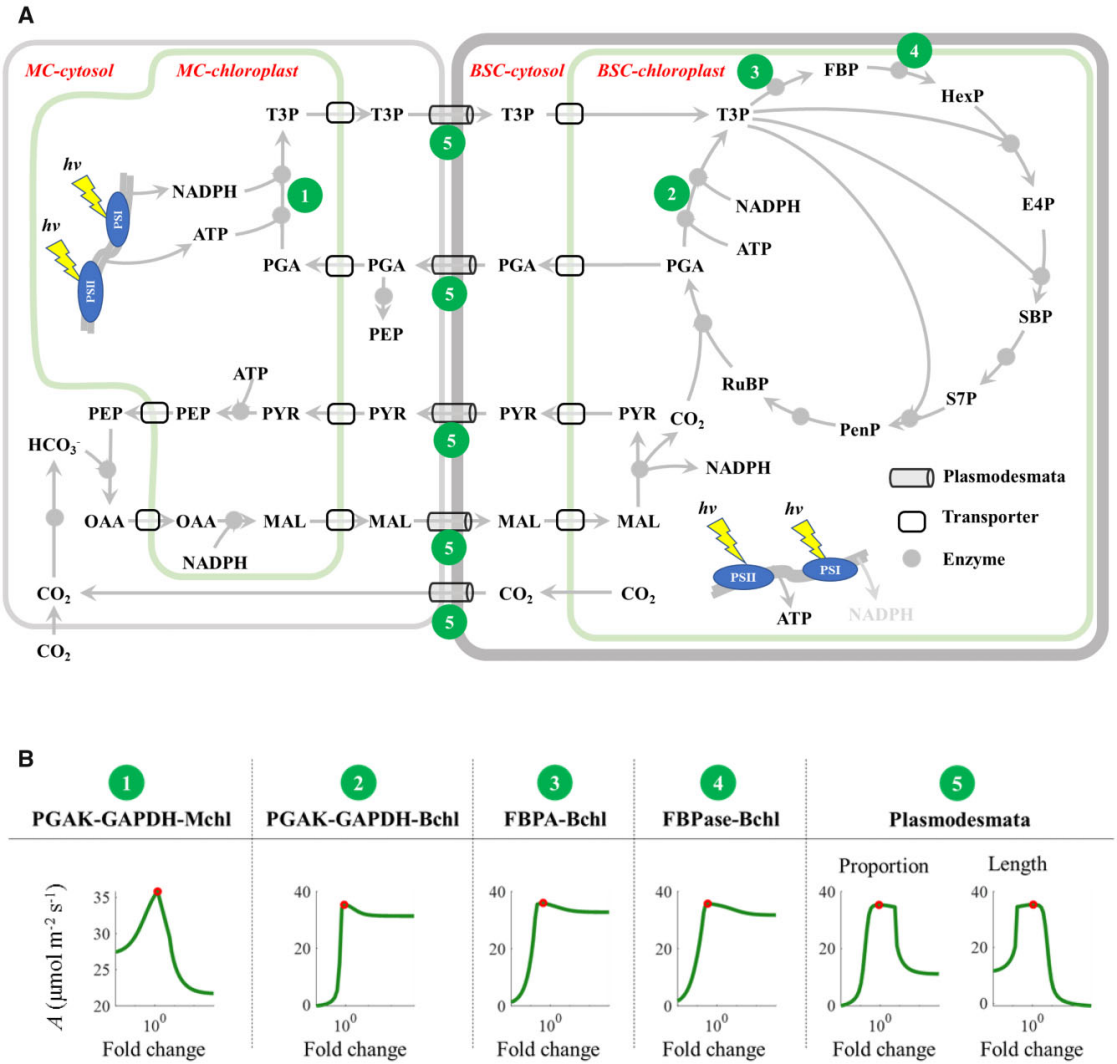
The metabolic processes and their corresponding parameters generating biphasic responses of photosynthetic rates to changes in their value in the NADP-ME type C_4_ photosynthesis. In (A), MC represents mesophyll cell, and BSC means bundle sheath cell. In (B) only steps whose *V*_max_ or feature parameters with biphasic impacts on photosynthetic rates are listed. Mchl indicates that the metabolic process occurs in the chloroplast or membrane of the MCs; Bchl indicates that the process occurs in the chloroplast or membrane of the BSCs; the plasmodesmata proportion represents the ratio of the plasmodesmata to the area of the BSC–MC interface, and its length represents the total cell wall thickness of both MC and BSC cell wall. The *x* axis represents the fold change of a parameter normalized against the parameter value resulting in the maximum net photosynthetic rate.

We used a dynamic systems model for the NADP-ME type C_4_ photosynthesis for the analysis (Wang et al., 2014a, 2014b). We first simulated the effects of increasing values of individual parameters on photosynthetic rates (*A*). Altogether 38 parameters in this model (36 *V*_max_ of enzymatic reactions and 2 parameters associated with plasmodesmata) were perturbed (Figure 2;  Supplemental Figure S1). Our results show that 6 out of 38 parameters have biphasic effects on the photosynthetic rates (Figure 2). These parameters include the maximal velocities of four enzymatic reactions and two parameters related to plasmodesmata. Following Zhao et al. (2020), we named enzymes producing biphasic responses as elements producing biphasic response (EPBR). The EPBR enzymes identified are PGAK-GAPDH in either chloroplast in MC (Mchl) or the Bchl, FBP aldolase, and FBPase in Bchl (Figure 2, A and B). The other 32 parameters produce nonbiphasic responses (Supplemental Figure S1). There are three types of nonbiphasic responses with the increase in enzyme activities: (1) first increase then a plateau; (2) first decrease and then a plateau; and (3) nonresponsive (Supplemental Figure S1).

The efficiency of C_4_ photosynthesis is determined by both the CBC for RuBP regeneration and C_4_ cycle for CO_2_ pumping (Figure 1). Rubisco catalytic capacity is maintained at its default value in our univariable analysis for EPBRs, so the changes of chloroplastic [RuBP] and [CO_2_] in BSC are two factors contributing to the changes in photosynthetic rates in our perturbation experiments (Figure 1; Equation 8). Next, we analyzed the impacts of EPBRs’ activities on the limitations of chloroplastic [RuBP] and [CO_2_] in BSC to photosynthesis. Thereafter, we analyzed the biochemical mechanisms underlying the influence of EPBRs on the operations of the CBC and C_4_ cycle.

### Excess capacity of EPBR limits the efficiency of the RuBP regeneration cycle or the C_4_ cycle

**Figure 3:**
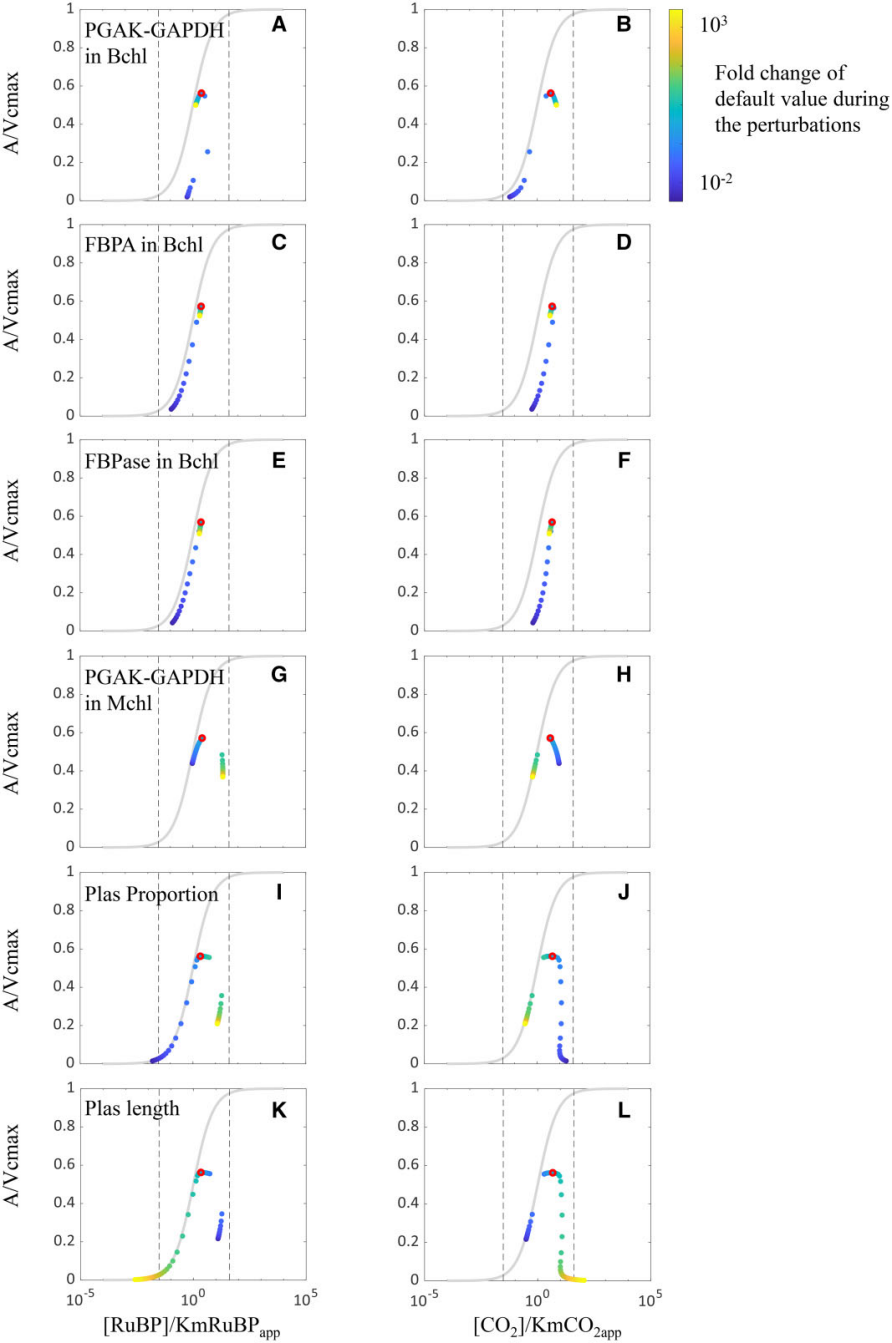
The influence of manipulating enzymes which have a biphasic impact on photosynthetic rates on [RuBP] and [CO_2_] and photosynthetic rates. The *y*-axis (*A/V*_cmax_) is the ratio of net photosynthesis to the Rubisco carboxylation capacity. The *x*-axis is the ratio of RuBP concentration around Rubisco to the apparent Michaelis–Menten constant of Rubisco for RuBP (subgraphs of A, C, E, G, I, and K in the left column), and the ratio of CO_2_ concentration around Rubisco to the Michaelis–Menten constant of Rubisco for CO_2_ (subgraphs of B, D, F, H, J, and L in the right column). The color of dots corresponds to fold change of the default value in a C_4_ photosynthesis model (Wang et al., 2014a, 2014b). The color from dark blue to yellow represents the fold change range from 0.01 to 1,000. The red dot marks a state which corresponds to the maximal photosynthesis. The perturbated parameters are the enzymatic capacities of the PGAK-GAPDH in the Mchl (A and B), of FBPA in the Bchl (C and D), of FBPase in the Bchl (E and F), of PGAK-GAPDH in the Bchl (G and H), for plasmodesmata proportion (I and J), and plasmodesmata length (K and L).

To elucidate the mechanistic basis of biphasic responses of NADP-ME type C_4_ photosynthesis to the increase in each EPBR capacity (Figure 2), we first determined the limiting factor for photosynthetic CO_2_ uptake rate, that is, whether it is [RuBP] or [CO_2_] or Rubisco activity that limits *A* under a particular perturbation. We plotted the enzyme–substrate relationship ([S]/KM versus v/*V*_max_) of Rubisco under a wide range of activities of each EPBR (Figure 3). Each subgraph is separated into three regions by two vertical dashed lines (Figure 3). For the left and right dashed lines, [S]/KM are 0.04 and 40 (Figure 3), respectively. These two values are based on previous studies (Fendt et al., 2010; Zhao et al., 2020). Three regions from left to right represent three limiting stages of Rubisco carboxylation (Figure 3), including the substrate limiting stage (the ratio of [S]/KM is ˂1 in the left region), enzymatic capacity limiting stage (the ratio of [S]/KM is >1 in the right region), and substrate and enzymatic capacity co-limiting stage (the ratio of [S]/KM is comparable to 1 in the middle region) (Figure 3; Fendt et al., 2010). The influence of perturbing activities of EPBRs on [RuBP], [CO_2_], and *A* (Figure 3, B, D, F, H, J, and L) are shown in Figure 3. During the perturbations, the default activity of each individual EPBR was changed from the range of 0.01- to 1,000-fold (the color bar in Figure 3). With the increase in activity of PGAK-GAPDH_Bchl_, the ratio of [RuBP]/KmRuBP_app_ increased first and then decreased (Figure 3A), meanwhile [CO_2_]/KmCO_2app_ kept rising (Figure 3B). With the increase in activity of FBPA_Bchl_ or FBPase_Bchl_, ratios of both [RuBP]/KmRuBP_app_ (Figure 3, C and E) and [CO_2_]/KmCO_2app_ (Figure 3, D and F) increased first and then decreased. With the increase in activity of PGAK-GAPDH_Mchl_, the ratio of [RuBP]/KmRuBP_app_ kept increasing (Figure 3G), meanwhile [CO_2_]/KmCO_2app_ kept decreasing (Figure 3H).

Plasmodesmata area proportion and plasmodesmata length are two factors determining the diffusion capacity of plasmodesmata between MC and BSC (Wang et al., 2014a, 2014b). According to the diffusion equation as in (Wang et al., 2014a, 2014b), the metabolites diffusion capacity between BSC and MC through plasmodesmata is positively associated with the proportion of plasmodesmata in the BSC–MC interface and is negatively associated with plasmodesmata length. In our in silico perturbation experiments, we increased the diffusion capacity of plasmodesmata, either by increasing plasmodesmata area proportion (as shown by the gradual change of color in Figure 3, I and J from blue to yellow) or by decreasing plasmodesmata length (as shown by the gradual change in color in Figure 3, K and L from yellow to blue). Results show that, with the increase in plasmodesmata diffusion capacity, the ratio of [RuBP]/Km_RuBPapp_ keeps increasing (Figure 3, I and K) and [CO_2_]/KmCO_2app_ decreases (Figure 3, J and L) in general.

Figure 3 shows that, excess activity of PGAK-GAPDH_Bchl_ inhibited the RuBP regeneration (Figure 3A); excess activity of FBPA_Bchl_ or FBPase_Bchl_ decreased the RuBP regeneration (Figure 3, C and E) and CO_2_ concentration (Figure 3, D and F); and excess capacity of PGAK-GAPDH_Mchl_ decreased the CO_2_ concentration (Figure 3H). Our earlier study showed that the high diffusion ability of plasmodesmata increases the leakage of CO_2_ from BSC to the MC and hence prevents the increase of CO_2_ concentration in the BSC (Wang et al., 2014a, 2014b). Consistent with previous results, increasing diffusion capacity of plasmodesmata, either through increasing plasmodesmata area proportion (yellow dots in Figure 3I) or decreasing plasmodesmata length (dark blue dots in Figure 3L), resulted in a lower [CO_2_] and decreased photosynthetic CO_2_ uptake rate (Figure 3, I and L).

In this study, when we conducted the perturbation analysis, we changed activities for enzymes individually, that is, the values of other parameters were not altered except the perturbed parameter. Therefore, the influences on metabolic fluxes should only be due to the changes in metabolite levels.

### A suitable PGAK-GAPDH activity in MCs is required for efficient NADP-ME C_4_ photosynthesis

**Figure 4:**
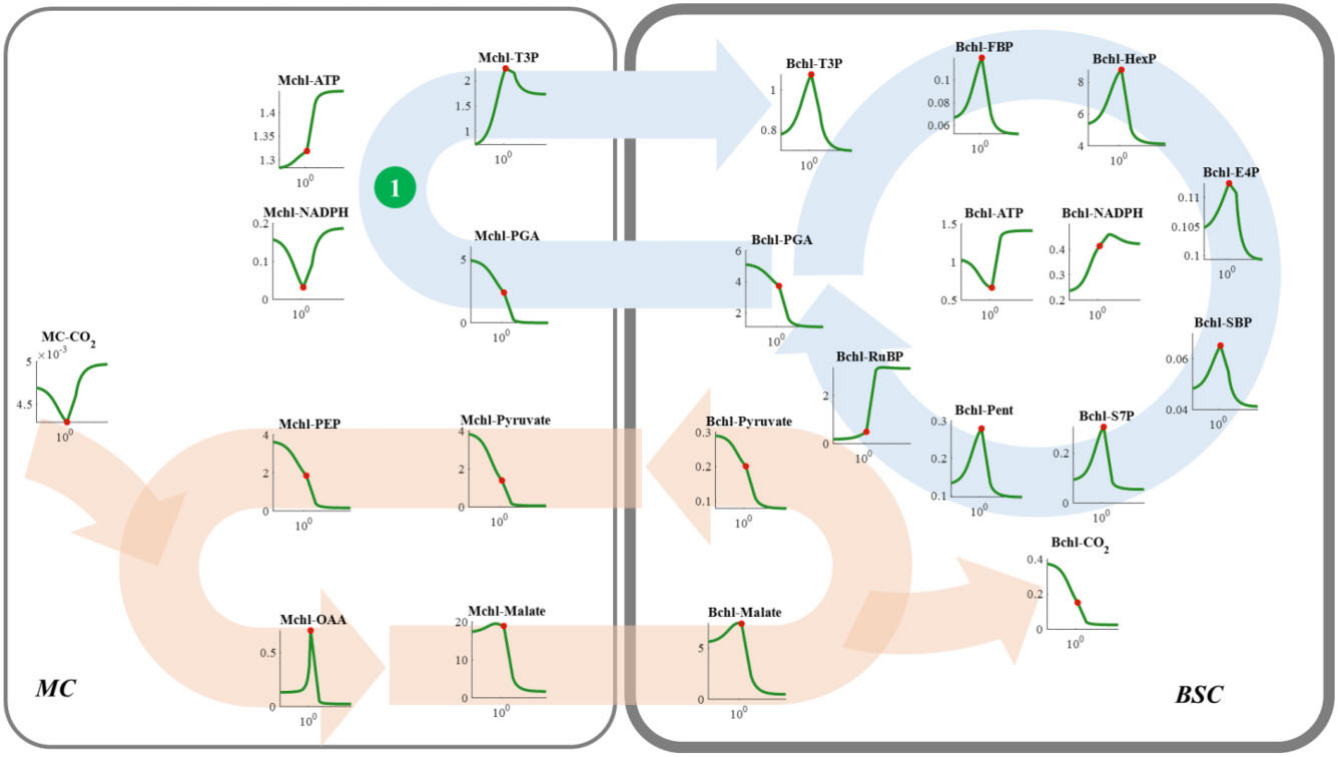
Response curve of metabolite levels in NADP-ME type C_4_ photosynthesis to changes in the *V*_max_ of PGAK-GAPDH in the MCs. The dark green circle numbered ① indicates the location of the PGAK-GAPDH catalyzed reaction in the MC; the circle with light blue curve arrow indicates the CBC; the circle with orange curved arrow indicates the CO_2_ concentrating mechanism. The *x*-axis represents the normalized fold changes of the parameter with the 1 representing the parameter resulting in the maximal photosynthetic rate. The *y*-axis represents the metabolite concentration in millimolar. The red dot marks a state which corresponds to the maximal photosynthesis.

To explore why a suitable PGAK-GAPDH activity in Mchl is needed for an efficient NADP-ME type C_4_ metabolism, the steady-state concentrations of individual metabolites in the CBC and CCM are plotted against the V_max_ of Mchl PGAK-GAPDH (Figure 4). We first considered the concentrations of RuBP and CO_2_ in Bchl. Before the maximal photosynthetic CO_2_ uptake rate (Figures 2, B and 4) is reached, increasing Mchl PGAK-GAPDH increased the RuBP concentration, while it decreased the CO_2_ concentration in the Bchl (Figure 4). This indicates that the rising phase of photosynthesis (Figure 2B) is caused by the increased RuBP concentration (Figure 3, G and H). As MC PGAK-GAPDH can translocate ATP and NADPH from MC to BSC for CO_2_ assimilation (Hatch and Kagawa, 1976), increasing its activity released the accumulation of PGA in both cell types and increased the pool size of the CBC intermediates in the BSC and consequently increased the RuBP accumulation (Figure 4). After *A* reached its maximal rate, further increase in MC PGAK-GAPDH activity decreased the CBC metabolite concentrations except RuBP, and decreased the CO_2_ level in the BSC (Figure 4). These simulations suggested the decreased photosynthesis (Figure 2B) was due to the decreased CO_2_ level (Figures 3, G and H and 4). In the decreasing phase, concentrations of all metabolites related to CCM were reduced. These perturbations for MC PGAK-GAPDH capacity suggested that the NADP-ME type C_4_ photosynthesis switched from RuBP regeneration limitation to CO_2_ concentration limitation with increase in the MC PGAK-GAPDH capacity.

### Suitable PGAK-GAPDH activity in BSCs is required for efficient NADP-ME C_4_ photosynthesis

**Figure 5:**
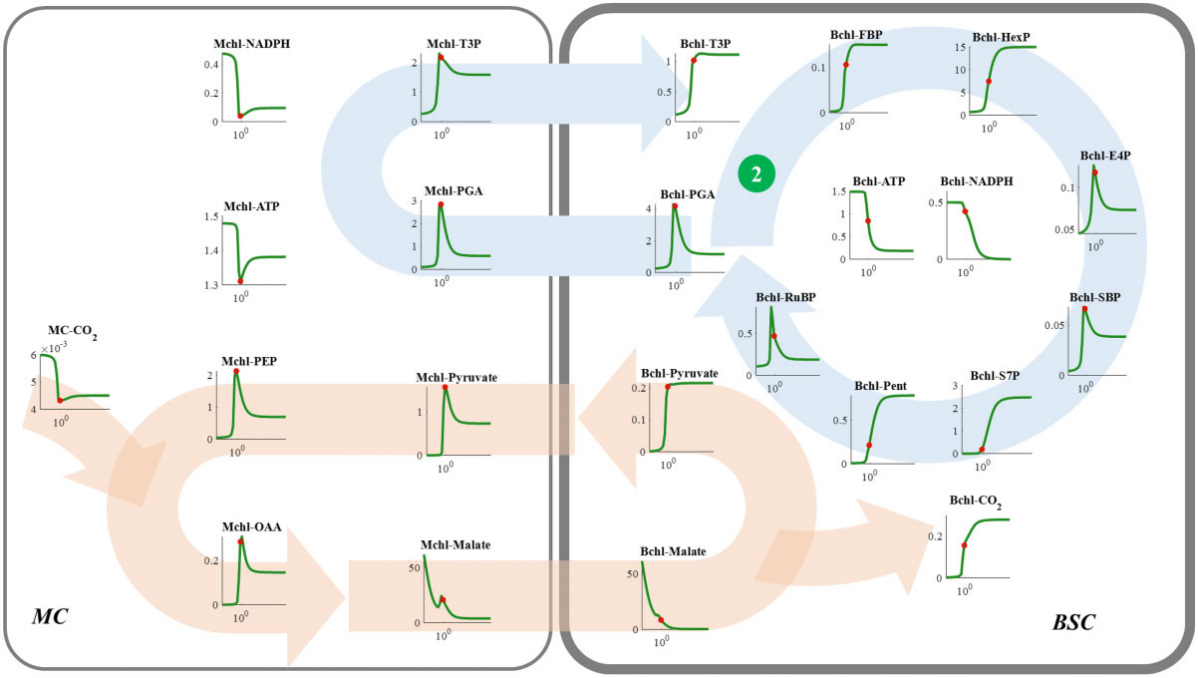
Response curve of metabolite levels in NADP-ME type C_4_ photosynthesis to the *V*_max_ of PGAK-GAPDH (The dark green circle numbered ②) in the BSC. See figure legend for Figure 4 for more details.

PGAK can coordinate the ATP consumptions in the CBC (Zhao et al., 2020). Consistent with this role in C_3_ photosynthesis, simulations with the dynamic model of NADP-ME type C_4_ photosynthesis shows a similar response of the CBC to the increased PGAK-GAPDH in the Bchl (Figure 5; Zhao et al., 2020). When the Bchl PGAK-GAPDH was lower than the optimum, with a decrease in its activity, the CBC efficiency gradually decreased and concentrations of all metabolites in the CBC were decreased. On the contrary, the concentrations of ATP and NADPH in both BSC and MC were increased (Figures 2, B and 5). However, when PGAK-GAPDH activity was higher than the optimum, ATP and NADPH contents in Bchl rapidly decreased and metabolites between PGAK-GAPDH and PRK catalyzed reactions (such as T3P, FBP, HexP, S7P, and Pent) accumulated (Figure 5). Both inadequate and excessive capacities of Bchl PGAK-GAPDH can decrease the RuBP concentration (Figure 5), resulting in a RuBP limitation for Rubisco carboxylation (Figure 3A) and consequently decreased *A* (Figure 2B;Zhao et al., 2020). Additionally, when the Bchl PGAK-GAPDH enzymatic capacity was insufficient, malate level increased (Figure 5), however, the concentrations of metabolites involved in the CCM, that is, pyruvate, phosphoenol pyruvate (PEP), and oxaloacetate (OAA), and also the Bchl CO_2_ concentration decreased. These results suggest that inadequate PGAK-GAPDH catalytic capacity decreased RuBP regeneration, repressed malate decarboxylation catalyzed by Bchl NADP-ME, and decreased the donor concentration for CO_2_ fixation by PEPC in MC (Figure 5). Although a Bchl PGAK-GAPDH catalytic capacity higher than the optimum reduced the contents of pyruvate (only in MC), PEP, OAA, and malate (in both cell types), the CO_2_ concentration in Bchl was maintained at about twice the level of that for the maximal photosynthetic CO_2_ uptake rate (red dot in Figure 5). Therefore, these perturbations for Bchl PGAK-GAPDH indicate the decrease of photosynthesis after the maximum PGAK-GAPDH (Figure 2B) can be attributed to the decreased RuBP concentration (Figure 5).

### Suitable FBPA and FBPase activities in the Bchl are required for efficient NADP-ME C_4_ photosynthesis

**Figure 6:**
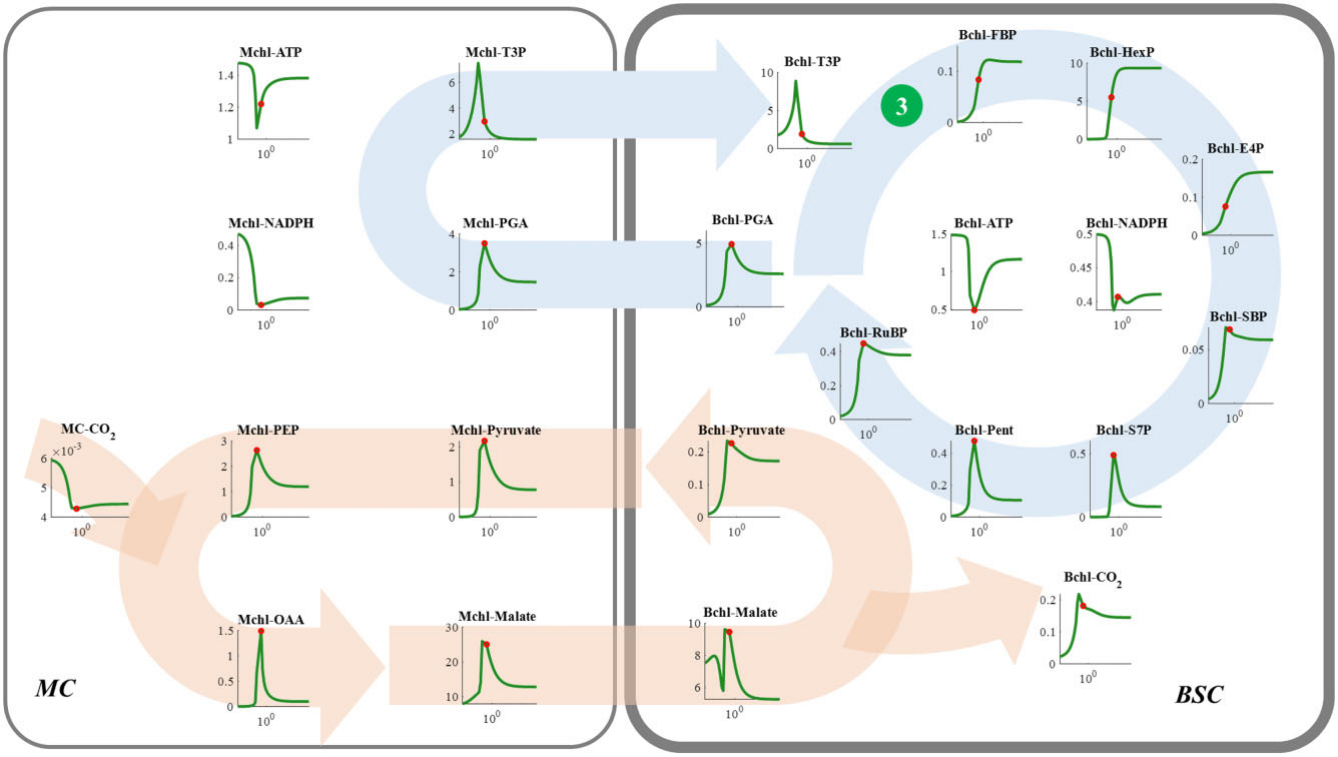
Responses of metabolite levels in NADP-ME type C_4_ photosynthetic metabolism to increase of the *V*_max_ of FBPA (The dark green circle numbered ③) in the BSC. See figure legend for Figure 4 for more details.

**Figure 7:**
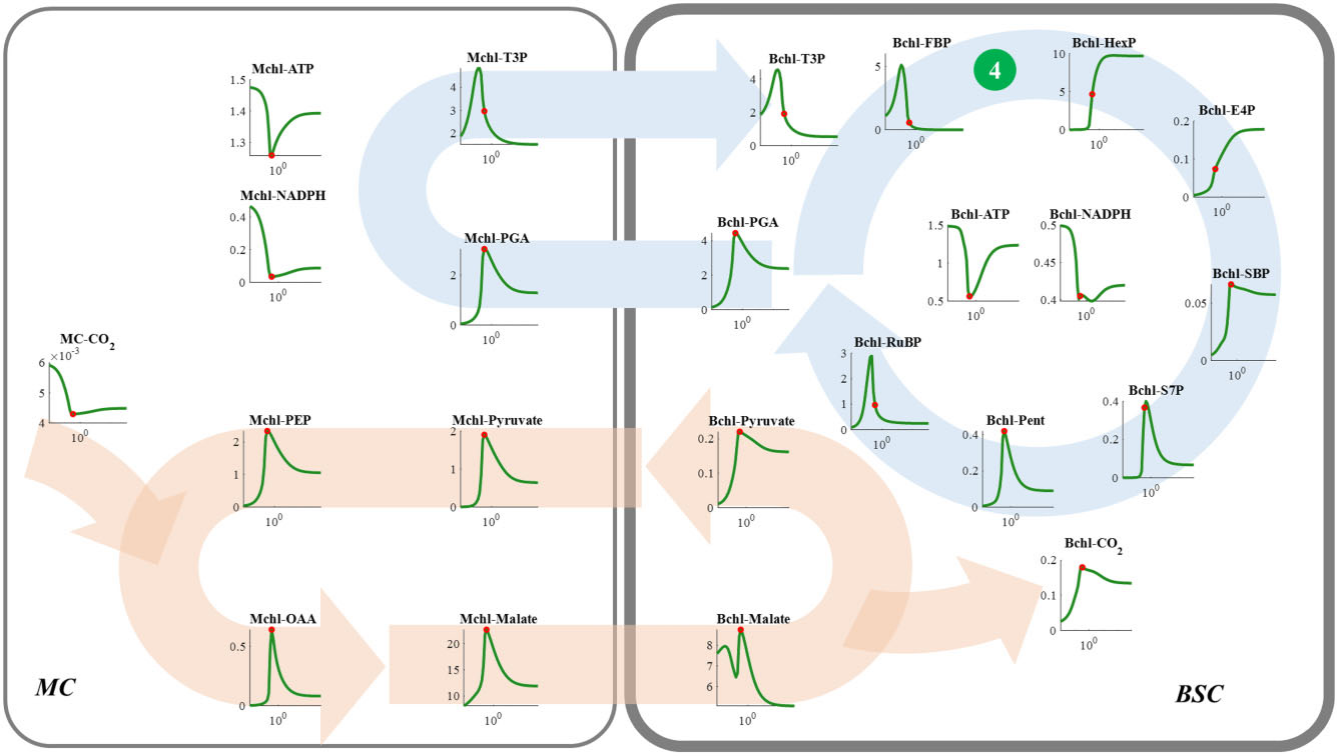
Responses of metabolite levels in NADP-ME type C_4_ photosynthetic metabolism to the changes in the *V*_max_ of FBPase in the BSC (the dark green circle numbered ④). See figure legend for Figure 4 for more details.

Similar to the function of FBPA and FBPase in C_3_ photosynthesis, increasing Bchl FBPA or FBPase activity increased the concentrations of the intermediates of the CBC (Figures 6 and 7; Zhao et al., 2020). However, further increase of their activities after the maximal photosynthesis led to a gradual decrease of photosynthesis, since increasing the conversion of T3P to produce FBP and HexP resulted in a shortage of T3P for other reactions in the CBC, ultimately leading to a decrease in the concentrations of other CBC intermediates, including SBP, S7P, Pent, RuBP, PGA, and T3P (Figures 6 and 7; Zhao et al., 2020).

Concentrations of all CCM metabolites decreased when the enzymatic capacity of Bchl FBPA or FBPase was higher than the level required for the maximal photosynthesis (Figures 6 and 7). Although the CO_2_ consumption rate declined after the maximal photosynthetic CO_2_ uptake rate, the CO_2_ concentration in the Bchl dropped (Figures 6 and 7). Therefore, the decrease of photosynthesis after the optimal FBPA and FBPase (Figure 2B) can be attributed to the combined effects of decreased RuBP concentration and the reduced CO_2_ concentration (Figures 6 and 7). It indicates that not only the efficiency of CBC, but also that of CCM can be inhibited by an excessive catalytic capacity of Bchl FBPA or FBPase.

### Suitable diffusion capacity of plasmodesmata between BSCs and MCs is required for efficient NADP-ME C_4_ photosynthesis

The plasmodesmata diffusion capacity is critical for intermediates translocation between BSC and MC, and hence affects the efficiency of C_4_ photosynthesis. Increasing the diffusion capacity generates a biphasic photosynthesis response curve of *A* (Figure 2B;Wang et al., 2014a, 2014b). Since increasing the plasmodesmata area proportion has the same effect on the plasmodesmata diffusion property as decreasing plasmodesmata length, we only analyzed the metabolic mechanisms of increasing plasmodesmata area proportion on C_4_ photosynthesis in this sub-session.

**Figure 8:**
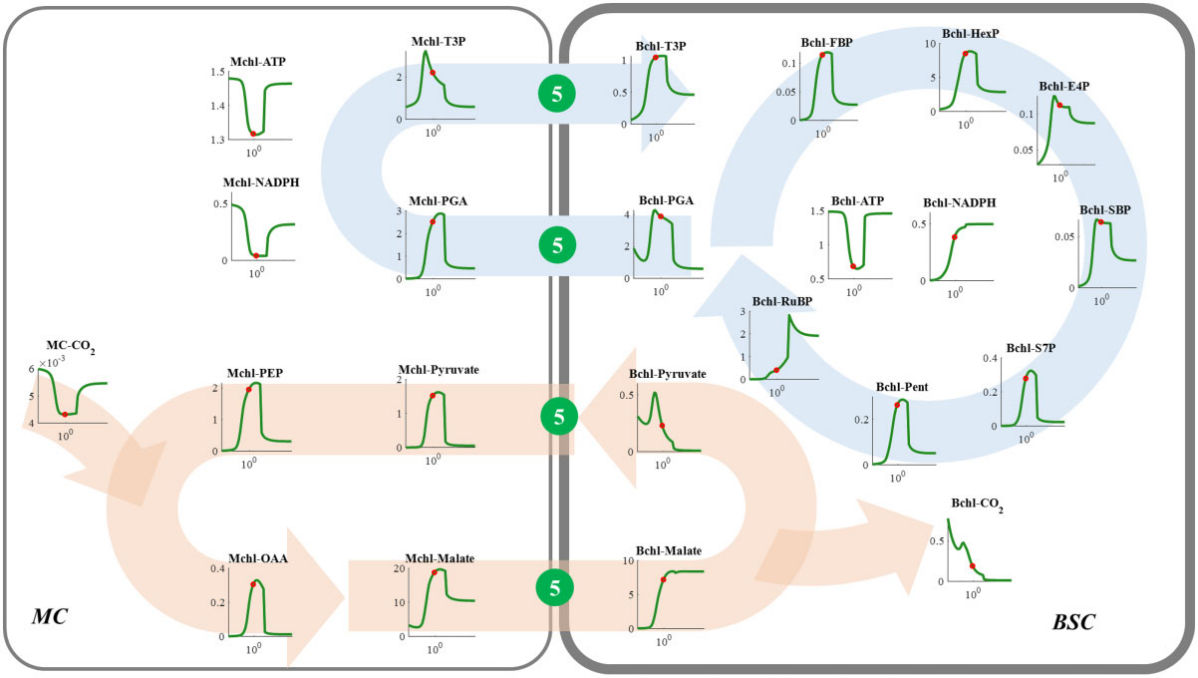
Responses of metabolite levels in NADP-ME type C_4_ photosynthetic metabolism to the Plasmodesmata (the dark green circle numbered ⑤) proportion. See figure legend for Figure 4 for more details.

With an increase in plasmodesmata area proportion, the RuBP concentration increased (Figure 8), and the BSC CO_2_ concentration decreased (Figure 8). This indicates that, before the plasmodesmata area proportion reached an optimum, an increase in photosynthesis with greater plasmodesmata area proportion resulted from an increased RuBP concentration (Figures 3, I and 8). Under these conditions, the limited plasmodesmata diffusion capacity constrained the translocation of intermediates, for example, the movements of PGA and pyruvate from BSC to MC, and the movements of T3P and MAL from MC to BSC. This led to the inhibited capacity of C_3_ and C_4_ shuttles between MC and MSC (Figure 8), and ultimately decreased NADPH provision for driving the RuBP regeneration in BSC. Although levels of CO_2_ and ATP were high in Bchl under these conditions, the intermediate levels in the downstream of PGA reduction and RuBP regeneration in the CBC were low (Figure 8), which ultimately limited NADP-ME type C_4_ photosynthetic efficiency (Figure 2).

However, a further increase in the plasmodesmata area proportion after the optimum led to decreased photosynthesis (Figure 2). Different from the reasons above, the inhibited photosynthesis at a high plasmodesmata area proportion was not caused by the changed level of RuBP in BSC (Figures 3, I and 8), but rather the changed CO_2_ levels (Figures 3, J and 8); this is because RuBP levels at high plasmodesmata area proportion conditions were always larger than those under the optimum condition (Figure 8). The excessive plasmodesmata area proportion, however, resulted in a decreased CO_2_ concentration and low concentrations of other intermediates in the CBC, except ATP and NADPH (Figure 8). Concurrently, levels of all the metabolites in the C_4_ cycle were also decreased when the plasmodesmata area proportion was higher than the optimum, except for malate levels in BSC (Figure 8).

### Changes in metabolite levels in the C_3_ metabolism affect CCM metabolism through influencing PGA concentration and redox levels

When we perturbed the enzyme activities in the C_3_ metabolism, PGA concentrations in the Mchl were affected by PGAK in the Mchl (Figure 4) and enzymes of the CBC in the Bchl (Figures 5–7). When the PGA concentration was perturbed, we found that the PEP concentrations have the same pattern of changes as that of Mchl PGA (Figures 4–8) in all simulations.

In some perturbations (Figures 5–8), both PGA and PEP concentrations decreased. However, malate accumulated to a high level and the CO_2_ concentration around Rubisco is low (Figures 5–8), which indicates the CCM is mainly limited by a low decarboxylation of NADP-ME. Since the enzyme activity and kinetic properties of NADP-ME were not changed in our perturbations (Figures 4–8), the decreased NADP-ME flux could only be caused by decreased substrate concentrations. Considering that the malate level was high, the limitation is caused by a low NADP^+^ (Hatch and Kagawa, 1976). In our model, the total content of NADPH and NADP^+^ is assumed as a constant in the Bchl ([NADP^+^] + [NADPH] = 0.5 mM; Wang et al., 2014a, 2014b). In our simulation, the high malate concentration and low CO_2_ concentration in the Bchl were accompanied by a high NADPH concentration (near to 0.5 mM; Figures 5–8) and low NADP^+^ level. Therefore, a suitable redox potential in the Bchl is required for malate decarboxylation in the Bchl.

## Discussion

**Figure 9:**
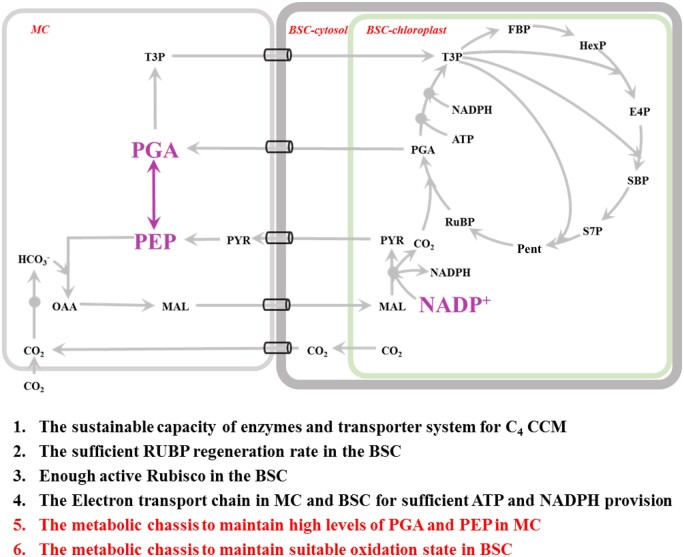
The requirements for an efficient NADP-ME type C_4_ photosynthesis. In addition to the known elements for an efficient NADP-ME type C_4_ photosynthesis (1–4 in black; Wang et al., 2014a, 2014b; von Caemmerer and Furbank, 2016), maintaining high concentrations of PGA and PEP in mesophyll chloroplasts and a high concentration of NADP^+^ in Bchl are also required to gain high C_4_ flux (5 and 6 in red). These two additional biochemical requirements are present in purple and with the larger font size in the graph.

Many features and required elements for an efficient NADP-ME type C_4_ photosynthesis (Wang et al., 2014a, 2014b; von Caemmerer and Furbank, 2016) have been discovered previously, which include sufficient capacities for enzymes and transporters involved in the C_4_ shuttle, sufficient capacity for RuBP regeneration, sufficient Rubisco capacity in the BSC, and sufficient electron transfer capacity in the MC and BSC for ATP and NADPH generation. These features are basic elements required for an efficient operation of C_4_ photosynthesis. This study quantitatively demonstrates that two additional mechanisms are required to effectively coordinate the C_3_ metabolism and C_4_ shuttle for a higher C_4_ photosynthetic efficiency (Figure 9). These two routes include (1) building up the PEP concentration through maintaining the PGA content in the MC to ensure substrate supply for the PEPC catalyzed reaction and (2) maintaining a suitable oxidation state in the BSC to enable an efficient malate decarboxylation via NADP-ME.

### Building up the PEP concentration by maintaining the PGA concentration in the Mchl is required for an efficient PEPC catalyzed carboxylation

The reactions catalyzed by FBPA, FBPase, and PGAK-GAPDH in the Bchl can influence RuBP regeneration in C_4_ photosynthesis (Figure 2), which is similar to their effects on C_3_ photosynthesis (Zhao et al., 2020). In C_4_ photosynthesis, not only the RuBP regeneration but also the CCM efficiency is affected by PGAK-GAPDH, FBPA, and FBPase (Figures 5–7). These results demonstrate the C_4_ cycle interacts closely with C_3_ metabolism and the coordination between them is critical for an efficient NADP-ME type C_4_ photosynthesis.

For the NADP-ME type of C_4_ CCM, the C_4_ cycle functions as a pump to concentrate CO_2_ around Rubisco in the BSC. Meanwhile, the reducing power is released during malate decarboxylation as NADPH which is stoichiometrically equal to the CO_2_ release rate. Two NADPH molecules are needed to fix one molecule of CO_2_ through the CBC in the BSC. The NADPH needed for CO_2_ assimilation in the BSC can be replenished from the BSC chloroplastic electron transport chain and the PGA/T3P shuttled from the MC (Hatch, 1987). The replenishment of BSC NADPH shuttled from MC rather than the direct generation within BSC is a strategy due to the decreased level of photosystem II (PSII) in Bchls (Hatch, 1987) and the limited capacity of electron transfer from water to NADP^+^ as observed in maize (Hatch and Kagawa, 1976). In a typical NADP-ME type C_4_ photosynthesis, during the processes of shuttling NADPH from MC, a portion of PGA produced by RuBP carboxylation is transported from BSC to MC, where it is reduced to T3P in the Mchl; thereafter, the T3P is transported back from MC to BSC and further as the intermediates of the CBC in the BSC (Huber and Edwards, 1975). Consistent with this, low concentration of PGA in the MC is unfavorable for ATP and NADPH replenishment from MC to BSC in the NADP-ME type of C_4_ photosynthesis (Hatch and Kagawa, 1976; Leegood and von Caemmerer, 1988; Wang et al., 2014a, 2014b).

Furthermore, PGA in the Mchl can not only function as the substrate of PGAK, but can also be reversibly converted to produce PEP by two reversible enzymes (Huber and Edwards, 1975). In our simulations, PEP and PGA contents always change synchronously under different perturbations (Figures 4–8). These results are consistent with those reported in previous studies in which PEP and PGA contents change synchronously under different environmental conditions in various C_4_ plants (Leegood and von Caemmerer, 1988, 1989). It indicates that the C_4_ cycle and C_3_ metabolism are closely linked due to the reversible conversions between PGA and PEP (von Caemmerer and Furbank, 2016). As a result, the disruption of coordination in CBC which leads to either a decrease in PGA production or the over-consumption of PGA can decrease PGA and PEP concentrations simultaneously, and ultimately suppressed CO_2_ fixation by PEPC and correspondingly decreased the downstream metabolites in the C_4_ cycle (Figures 2–8). With the interconversions between PEP and PGA, it seems difficult to build up the PEP content completely dependent on PEP regeneration through the C_4_ cycle. In other words, to support enough substrate PEP as the donor of CO_2_ fixation via PEPC, it is required to build up the high level of PEP in the MC through maintaining a high PGA concentration.

In the C_4_ rice engineering, the flux of CO_2_ fixation by PEPC was increased within C_4_ transgenic rice (Lin et al., 2020; Ermakova et al., 2021), but the flux of PEPC in transgenic rice was only 2% of that in maize (Ermakova et al., 2021). This might be the result of the low activity of PEPC (Lin et al., 2020; Ermakova et al., 2021) and the low PEP concentration in the transgenic rice plants (Fukayama et al., 2003; Ermakova et al., 2021). Both the content of PEP and PGA in rice are much lower than that in maize (Fukayama et al., 2003; Arrivault et al., 2019). Considering that the affinity of leaf PEPC for PEP in maize (*K*_m_ = 1.48 mM) is much lower than that in rice (*K*_m_ = 0.1–0.12 mM; Masumoto et al., 2010, 2015), besides the PEPC activity, gaining a high PEP concentration is also required for an efficient operation of C_4_ type PEPC. Therefore, it is needed to maintain a high PGA concentration in MC to support an efficient NADP-ME type C_4_ photosynthesis.

In a typical C_3_ MC, five-sixths of PGA is metabolized for RuBP regeneration and near a half of net produced PGA is used for starch synthesis (Szecowka et al., 2013). Different from that, in NADP-ME type C_4_ plants, except the PGAK, GAPDH, and TPI, the other CBC enzymes are mainly located in the Bchl (Schlüter and Weber, 2020), and starch synthesis is usually located in Bchl where the CBC is located (Lunn and Furbank, 1997). This localization of starch synthesis and most of the CBC cycle in the BSC may help maintain a high concentration of PGA in the MC, in addition to the recognized role of transferring reducing equivalent and ATP from MC to BSC (Wang et al., 2014a, 2014b). Consistently, our predictions show that further increasing activities of Aldolase and FBPase in MC cytosol to consume more T3P and PGA for sucrose synthesis can also decrease C_4_ photosynthesis (Supplemental Figure S1), as a result of their impacts on PGA, T3P, and PEP levels (Supplemental Figure S2) in the MC. In C_4_ rice engineering, to build up the PEP concentration through maintaining PGA concentration, downregulation of the whole fluxes of CBC and starch synthesis in the MC to reduce the consumption of PGA in MC might be important strategies.

### Maintaining a suitable redox status in Bchls is required for efficient NADP-ME photosynthesis

Another observation from this study is that CO_2_ may still become a limiting factor for photosynthesis even when the malate concentration is high (Figures 5 and 8), which reflects a limitation of malate decarboxylation by the availability of NADP^+^. In our simulations, a high level of malate is accompanied with an accumulation of NADPH in the Bchl (Figures 5 and 8), which is consistent with the previous study which proposed that the oxidation state of the chloroplast is the driver of NADP-ME catalyzed malate decarboxylation (Bräutigam et al., 2018).

The lack of NADP^+^ can lead to a low efficiency of malate decarboxylation and correspondingly a low efficiency of photosynthesis itself. To date, in the C_4_ rice engineering project, levels of all enzymes belonging to the NADP-ME CCM pathway have been successfully increased in transgenic rice, and this increases the fluxes of CO_2_ fixation by PEPC to produce aspartate and malate (Lin et al., 2020; Ermakova et al., 2021). However, the malate decarboxylation flux through NADP-ME is undetectable (Lin et al., 2020; Ermakova et al., 2021). A number of factors have been proposed to contribute to this lack of decarboxylation fluxes. First, this might be due to a low level of NADP-ME expression in transgenic plants (Lin et al., 2020; Ermakova et al., 2021). Second, lack of transporters required for the NADP-ME CCM and the incorrect location of enzymes might contribute to the lack of fluxes (Lin et al., 2020). Third, results from this study suggest that the lack of sufficient flux might also be due to a shortage of NADP^+^ for malate decarboxylation (Figures 5 and 8). This might be the case in the study of Tsuchida et al. (2001), who constructed transgenic rice plants with increased NADP-ME activity up to 30-fold of that in WT plants; however, the enhanced NADP-ME activity in rice resulted in photoinhibition and retarded plant growth (Tsuchida et al., 2001).

What might be the strategies to maintain a more oxidized redox status in BSC? First, mixed decarboxylation types exist in almost all the natural C_4_ plants (Furbank, 2011), which might contribute to establishment of an appropriate redox status (Stitt and Zhu, 2014; Wang et al.,2014a, 2014b). This agrees with the observation that, feeding exogenous aspartate increases the rate of light-dependent malate decarboxylation in the BSC of maize leaf without increasing NADP-ME activity (Chapman and Hatch, 1979). In addition to maintaining the suitable redox status, the mixed strategy can also decrease the demand for maintaining high concentrations of a particular C_4_ acid for an efficient C_4_ photosynthesis (Pick et al., 2011; Wang et al., 2014a, 2014b). Besides using a mixed pathway, in NADP-ME type C_4_ plants, the reduced PSII content in the BSC might be another strategy to generate a more oxidized status (Leegood et al., 1981; Nakamura et al., 2013; von Caemmerer and Furbank, 2016; Ermakova et al., 2020).

### Summary

In the current effort of either improving C_4_ photosynthesis or engineering C_3_ plants to perform C_4_ photosynthesis, the Kranz anatomy, the capacities of enzymes, transporters, diffusion across plasmodesmata, and electron transport chain are considered as the major elements for an efficient C_4_ CCM (von Caemmerer and Furbank, 2016; Ermakova et al., 2020). Evidence from this quantitative simulation study shows that, besides these known requirements, maintaining high levels of PGA in MC and a suitable oxidized status in the Bchl are also required features for an efficient NADP-ME type C_4_ CCM. Potential strategies in C_4_ leaves to maintain a high PGA level in the MC include (1) constraining the starch synthesis pathway into the BSC and (2) decreasing the fluxes of the whole CBC in MC. The potential strategies to maintain a suitable oxidized status in the BSC include the mixed decarboxylases, maintaining minimal or no PSII activity in the BSC, and other factors discussed previously (Stitt and Zhu, 2014). More experimental studies are still needed to test these predicted features on improving C_4_ photosynthetic efficiency and engineering C_4_ rice.

## Materials and methods

### Parameter perturbations

The dynamic model of NADP-ME type C_4_ photosynthesis based on the Michaelis–Menten equation constructed previously (Wang et al., 2014a, 2014b) was used as a basis for our analysis. The model includes the metabolic pathways related to the NADP-ME type CCM which includes the CBC in the BSC, sucrose synthesis in the MC and starch synthesis in the BSC, photorespiration and the NADP-ME type C_4_ shuttle process. The transporters located on the chloroplast membrane mediating metabolite translocation between chloroplast and cytosol, and transporters located on the plasmodesmata linking BSC and MC for metabolites diffusion are also included (Wang et al., 2014a, 2014b). In this study, all the initial maximum velocities of the enzymes were the default values assigned in the previous model (Wang et al., 2014a, 2014b), in which the values of parameters were collected mostly for maize (*Z.* *mays*). The main structure has been simplified and redrawn in this study (Figures 1 and 2, A). The default maximal velocities of all enzymes (*V*_max_(s) of 36 enzymes) and parameters of plasmodesmata, that is, the area proportion and length, were perturbated with a range of fold change between 0.01 and 1,000. In each simulation, we only perturbed one parameter with all other parameters maintaining their default values. All the simulations were performed using MATLAB (2012). The method of perturbations is the same as (Zhao et al., 2020).

## Supplemental data 

The following materials are available in the online version of this article.

**Supplemental Figure S1.** The response curves of photosynthesis to the fold changes of enzymatic capacity.

**Supplemental Figure S2.** The response curves of PGA, T3P, and PEP to the fold changes of enzymatic capacity.

## Supplementary Material

kiac051_Supplementary_Data

## Notes

H.L.Z., and X.G.Z. designed this project. H.L.Z. and Y.W. conducted the simulations. H.L.Z., M.J.L., and X.G.Z. performed the analyses. H.L.Z. and X.G.Z. wrote the paper. All the authors edited the manuscript.

The author responsible for distribution of materials integral to the findings presented in this article in accordance with the policy described in the Instructions for Authors (https://academic.oup.com/plphys/pages/general-instructions) is Xin-Guang Zhu (zhuxg@cemps.ac.cn).
