# Supplementary material for: Two major metabolic factors for an efficient NADP-malic enzyme type C4 photosynthesis
Source: Plant Physiol. 2022 Feb 15;189(1):84–98. doi: 10.1093/plphys/kiac051 (PMC9070817; doi:10.1093/plphys/kiac051)

Supplemental Figure

Figure S1. The response curves of photosynthesis to the fold changes of enzymatic capacity. Note: CA: Carbonic anhydrase; PEPC: Phosphoenolpyruvate carboxylase; NADP-MDH: Malate dehydrogenase ($NADP^+$); NADP-ME: NADP-Malic enzyme; PPDK: Pyruvate, phosphate dikinase; Rubisco: Ribulose-bisphosphate carboxylase; SBPA: Sedoheptulose-bisphosphate aldolase; SBPase: Sedoheptulose-bisphosphatase; Tka: Transketolase; TKb: Transketolase; PRK: Phosphoribulokinase; PGCAP: Phosphoglycolate phosphatase; GO: Glycollate oxidase; GGAT: Glutamate Glyoxylate aminotransferase; GDC: Glycine decarboxylase; SGAT: Serine Glyoxylate aminotransferase; HPR: Glycerate dehydrogenase; GLYK: Glycerate Kinase; $Aldolase_{MC}$: Fructose-bisphosphate aldolase in the mesophyll cell chloroplast; $FBPase_{MC}$: Fructose-bisphosphatase in the mesophyll cell chloroplast; UGPU: UTP-glucose-1-phosphate uridylyltransferase; SPS: Sucrose phosphate synthase; SPP: Sucrose phosphatase; PFK: 6-phosphofructo-2-kinase; F26BPP: fructose-2,6-bisphosp hate 2-phosphatase; GPA: Glucose-1-phosphate adenylyl transferase; Diphosphatase: inorganic diphosphatase; Starch Synthase: ADPG Pyrophospho-rylase; $ATPase_{MC}$: ATP synthase in the mesophyll cell chloroplast; FNR: Ferredoxin-NADP+ reductase; $ATPase_{BSC}$: ATP synthase in the bundle sheath cell chloroplast.

Supplemental Figure

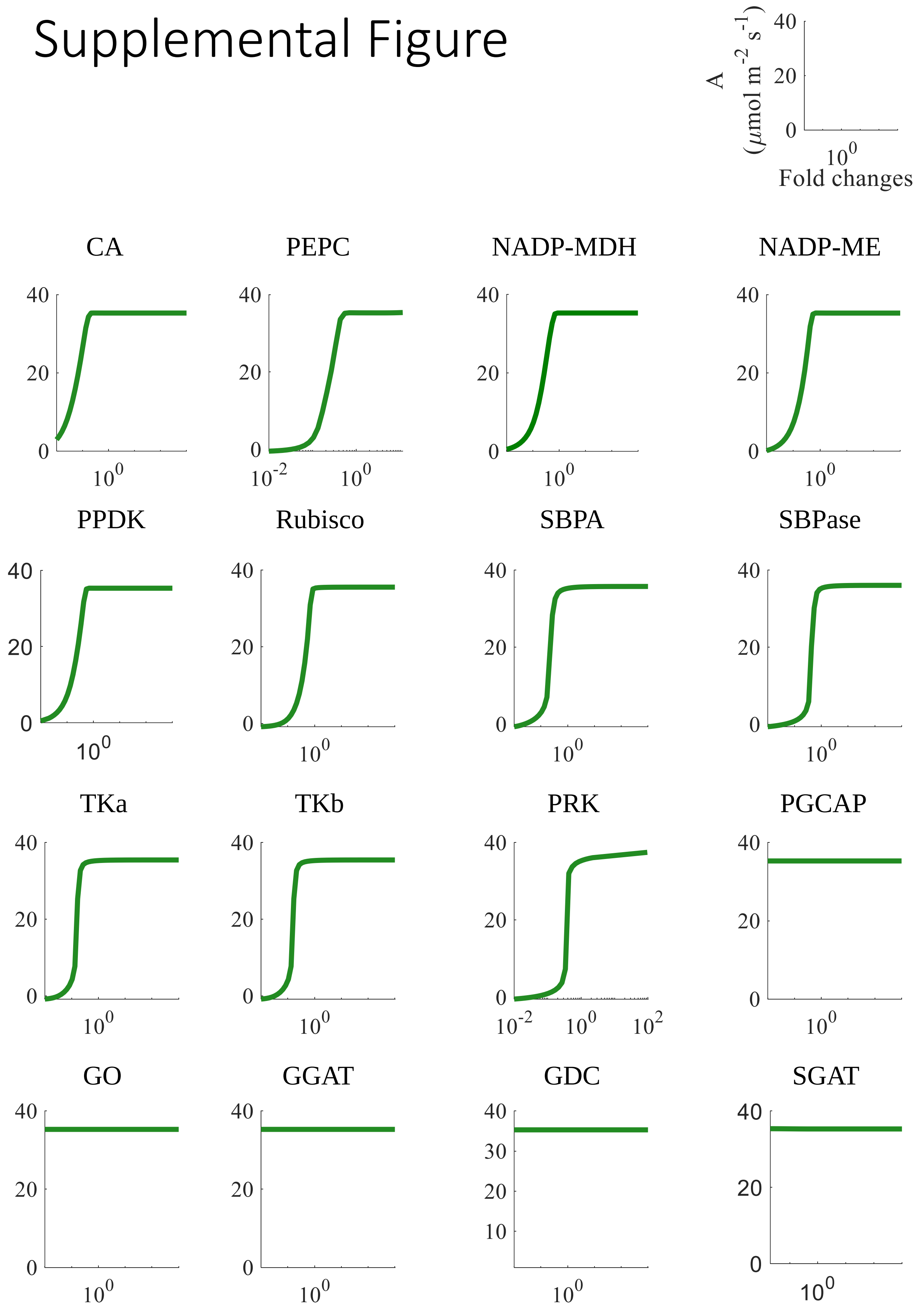

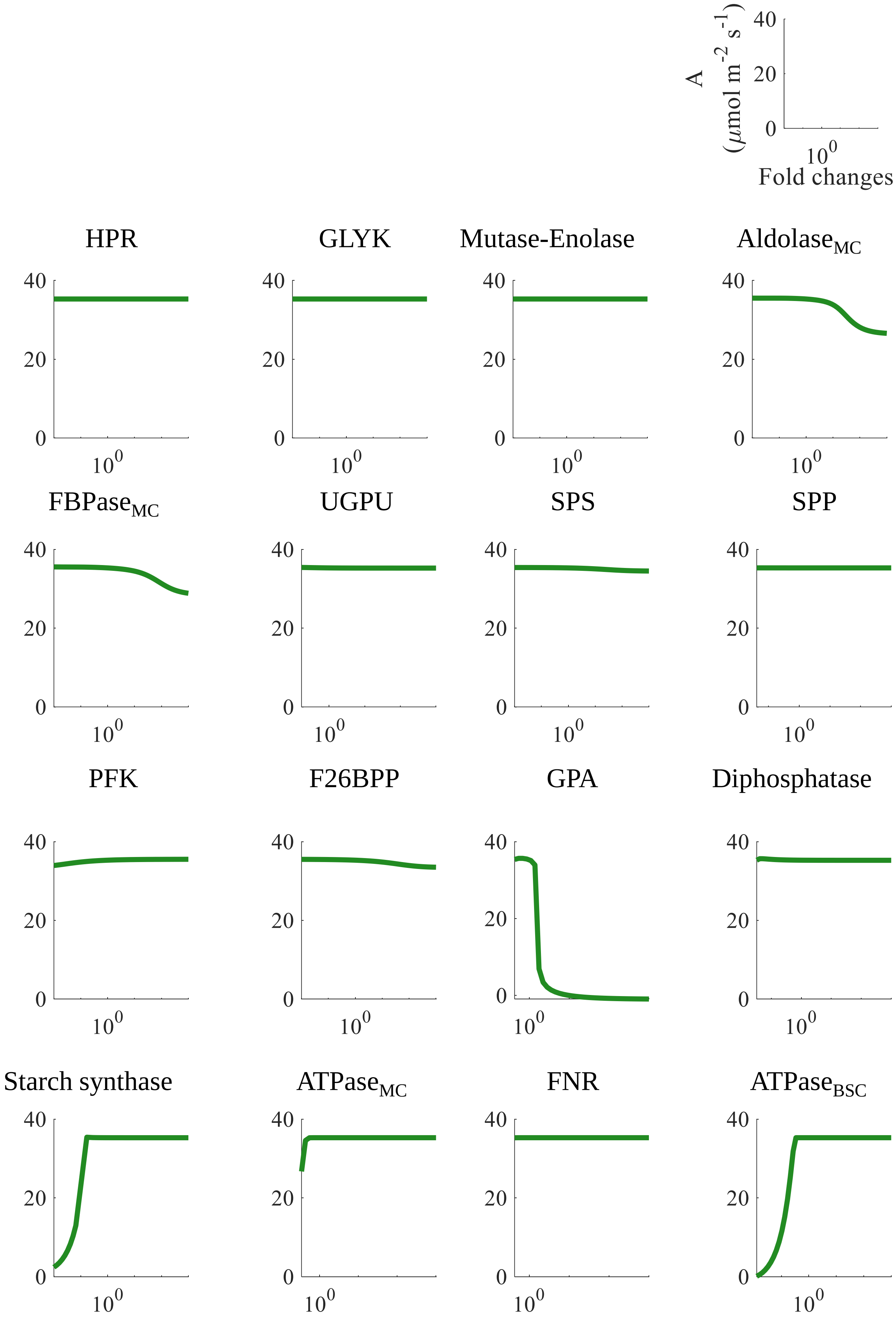

A (μmol m-2 s-1)
Fold changes
HPR
GLYK
Mutase-Enolase
Aldolase$_{MC}$
FBPase$_{MC}$
UGPU
SPS
SPP
PFK
F26BPP
GPA
Diphosphatase
Starch synthase
ATPase$_{MC}$
FNR
ATPase$_{BSC}$
40
20
0
10^0

Figure S1.

- Figure S2. The response curves of PGA (a and b), T3P (c and d) and PEP (e and f) to the fold changes of enzymatic capacities. $Aldolase_{MC}$ (a, c and e) and $FBPase_{MC}$ (b, d and f) are the enzymes of sucrose synthesis in mesophyll cells.

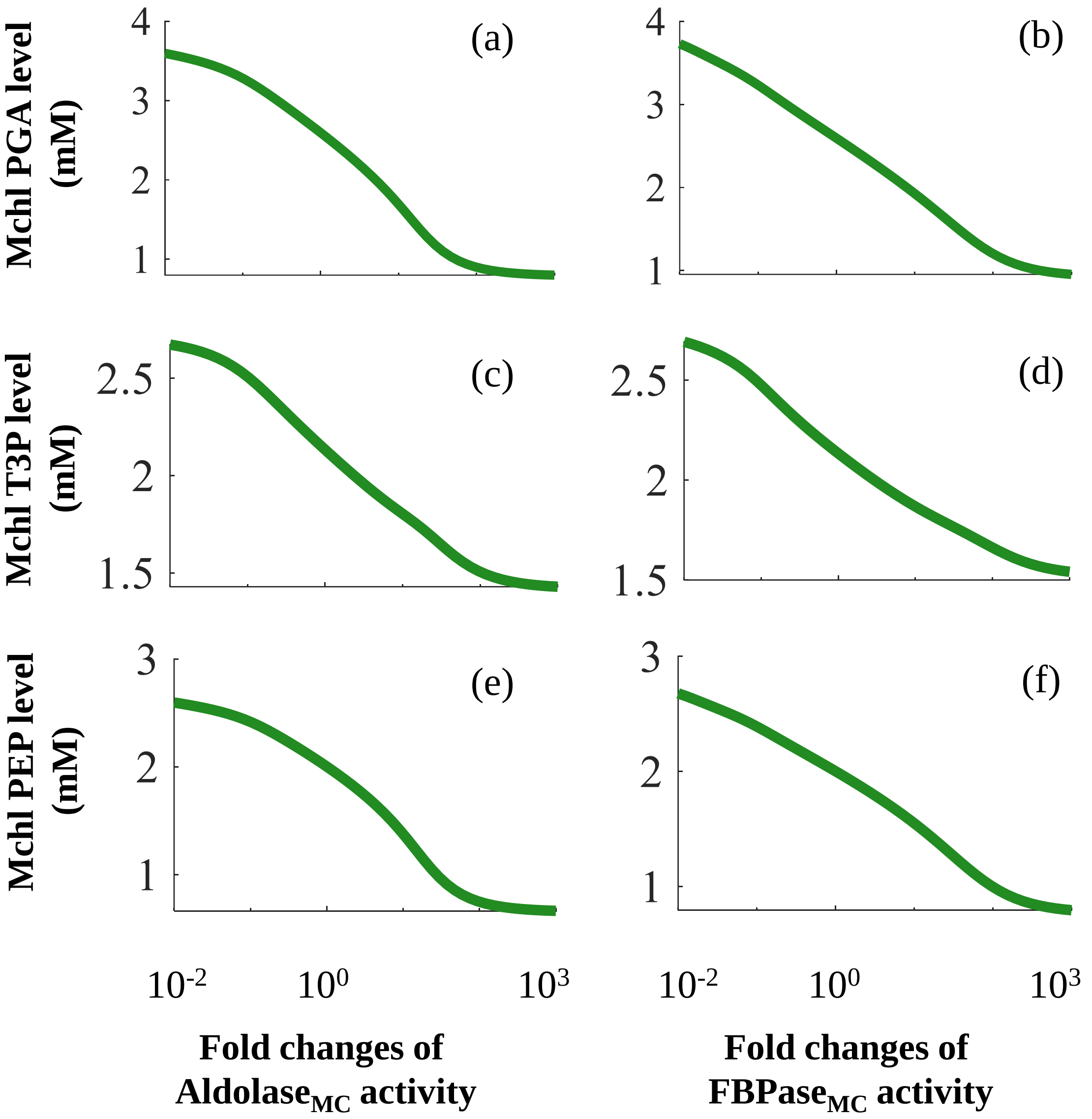

Supplement: kiac051_Supplementary_Data [file kiac051_supplementary_data.pdf]
